# Contact screening and risk factors for TB among the household contact of children with active TB: a way to find source case and new TB cases

**DOI:** 10.1186/s12889-019-7597-0

**Published:** 2019-09-18

**Authors:** Madeeha Laghari, Syed Azhar Syed Sulaiman, Amer Hayat Khan, Bandeh Ali Talpur, Zohra Bhatti, Naheed Memon

**Affiliations:** 1 Department of Clinical Pharmacy, School of Pharmaceutical Sciences, Universiti Sains Malaysia, 11800, Minden Penang, Penang, Malaysia; 2 School of Computer Science and Statistics, Trinity College Dublin, Dublin, Ireland; 3 College of Pharmacy, Liaquat University of Medical and Health Sciences, Jamshoro, 76090 Pakistan

**Keywords:** Children, Contact screening, Tuberculosis, Risk factors

## Abstract

**Background:**

Source case investigation, for children with tuberculosis (TB), is conducted to establish the source of infection and to minimize the extent of on-going transmission from infectious persons in the community. The aim of the study was to evaluate the secondary TB cases and to investigate the risk factors in developing TB among the household contacts (HHC) of children with active TB.

**Methods:**

A prospective cross-sectional study was conducted where 443 caregivers, of 508 children with active TB receiving treatment, were interviewed using a structured questionnaire. Logistic regression analysis was used to examine the risk factors for TB.

**Results:**

A total of 2397 family members at the median of 5 persons were recorded. Of these, 223 (9.3%) were screened on symptoms basis and 35 (15.7%) of these contacts were diagnosed with TB. Multivariate analysis revealed HHC with TB (OR = 15.288, 95% CI: 5.378–43.457), HHC with smoking (OR = 7.094, 95% CI: 2.128–23.648), and contact of > 18 h with TB individual (OR = 4.681, 95% CI: 1.198–18.294) as statistically significant risk factors of TB among the HHC.

**Conclusion:**

With the current system of contact screening for TB, only 9.3% of all HHC were screened. The low rates of contacts screened are possibly a repercussion of the passive nature of the program, which mainly depend on distinctive clinical symptoms being experienced by the contacts. Strategies are required to certify adherence with contact screening among children with active TB and to critically consider the factors responsible for TB transmission.

## Background

WHO has defined systematic screening for active tuberculosis (TB) as “the systematic identification of people with suspected active TB, in a preset target group, using tests, examinations or other procedures that can be applied swiftly” [1]. According to the published literature, a single pulmonary TB (PTB) patient can infect 10 to 15 persons on the average [2], having close contact, with in a community whereas 90% of the TB transmission in the community is due to sputum smear positive [3, 4]. Household contacts (HHC) are highly susceptible to acquire TB infection from the index cases because of their close proximity. The goal of contact tracing and their screening for TB could lead to the detection of additional cases of TB, maximizing the impact of case detection and effective treatment [5, 6]. The prevalence of TB disease is particularly soaring among children who are close contacts of a TB patient. Hence, screening children as contacts is generally recommended, though practiced in rarity [1].

Contacts of TB patients are at the greater risk of obtaining either TB infection or TB disease, depending on factors such as type of contact infectiousness of source case and environmental characteristics. In addition, host-related factors (age and immunology) additionally intervene with the likelihood of the patient getting to be infected or ill [7]. Enhanced learning of risk factors determining the possibility of contacts being infected with TB would reinforce contact investigations by enhancing prioritization so high-risk contacts can be focused on first.

Although screening and managing TB contacts are recommended, but not often done in low-income countries [8–10]. A shortage of tuberculin, absence of chest X-ray (CXR) machines, lack of staff skill to figure out diagnostic results, minimum of 2 appointments needed to complete screening, transport and time costs for the patients and their families and high workload over healthcare workers have all been identified as barriers to screening [11]. Information on the contribution of routine contact investigations to early TB case detection is scarce in these countries.

Pakistan ranks fifth amongst the 22 high burden TB countries with TB incidence rate of 270 per 100,000 population and prevalence of 341 per 100,000 population; however, only 62% of these cases are detected and reported to the National TB Program (NTP) [12]. In Pakistan, passive case finding approaches were mostly used previously for TB case detection. Although, recently the NTP recommends use of active case finding in targeted population in order to enhance case finding [13], however, the practice is still limited in number of settings providing *Directly Observed Treatment Short* Course (DOTS) and only applied in screening contacts of TB adults. Nevertheless, there is very limited information on the value of contact investigation and approaches used in HHC screening of children with active TB in high incidence settings like Pakistan. The present study was aimed to evaluate the secondary TB cases and to investigate the risk factors in developing TB among the HHC of children with active TB.

## Methods

### Study settings and design

A cross-sectional study was conducted in five public health units of Hyderabad, Jamshoro and Matiari districts of Sindh province namely Liaquat University of Medical and Health Sciences Civil Hospital, Hyderabad (LUMHS-CHH), Shah Bhitai Government Hospital, Latifabad, Hyderabad (SBGH-LH), Sindh Government Hospital Qasimabad, Hyderabad (SGH-QH), Institute of Chest Diseases Kotri, Sindh (ICDK), Syed Baqadar Shah Taluka Hospital, Matiari (SBS-THM), where children ≤ 14 years were registered and their treatment outcomes were studied during previous phase of study [14]. In this study, our study population was comprised of HHC of children with active TB. The current study is the part of the previously published study [14] conducted at above mentioned public health units.

A structured questionnaire consisting of patient characteristics and demographic information was used (Additional file 1). Patient related information contained age, gender, type of TB while demographic factors included residential features, contacts with history of TB, contacts under treatment of TB at the time of study, and relationship of contacts with index case. All study procedures including data abstraction from clinical records, management of structured interviews, counselling and testing for HIV and TB, were performed after obtaining verbal informed consent from the caregivers and HHCs. All the information was collected based on NTP guidelines [13].

All caregivers of registered index cases [14], who were able to communicate, understand the questions and provide oral informed consent, were eligible for inclusion. Interviews were held using structured questionnaires in the waiting room during a period of about 20 min. Of 508 caregivers, 443 accepted to participate in study. Those who refused to participate, 42 (64.6%) reported that they have no time to participate whilst 23 (35.4%) gave no explanation about. An index TB case in our study was defined as the children aged ≤ 14 years who presented and registered for TB treatment at the above said health units. An index case at these centers is diagnosed as having TB by considering suggestive clinical features, the history of contact, positive TST (≥ 10 mm was considered positive) [15], scoring charts (suggested by the Pakistan Paediatric Association) and evidence of TB in chest X-ray (CXR), sputum microscopy (mostly children greater than 7 years) for pulmonary TB (PTB). Culture and Xpert MTB/RIF assays were used as add-on tests and specifically performed to exclude resistant TB We were unable to collect induced sputum or gastric aspirates from children who could not produce sputum, as these facilities are not frequently used at the study site [14]. For EPTB, diagnostic tests were performed depending on the sites involved [13].

HHC was defined as any person who had lived in the same house as the index case for at least 3 months and had slept in the same house for on average at least 4 nights per week, for a period of at least 3 months leading up to the time of diagnosis of the index case. Secondary cases were defined as a HHC who was diagnosed to have TB in the interval (days) between the day start of treatment of the index case and the day of interview of the household [16]. Source case was any HHC with infectious TB (usually sputum smear or culture-positive) during a period when the child could have been exposed.

At the time of enrollment of an index case, contacts (children and adults) presented with symptoms suggestive of PTB (cough ≥ 2 weeks, fever and weight loss) and extra-pulmonary TB (EPTB) were asked to come to hospital for screening. Participants were requested to bring children < 5 years to the hospital whether symptomatic or asymptomatic. HHC who were willing for screening were registered at the study sites and their demographic details were noted. HHCs were screened using a CXR and evaluated for any symptoms suggestive of TB. Any abnormal finding on CXR with symptoms suggestive of TB were referred for sputum microbiological examination including smear and Xpert MTB/RIF. Contacts with an abnormal CXR and the absence of symptoms, were given a course of antibiotics and a repeat CXR was performed two weeks later to exclude persisting abnormality. However, all the asymptomatic contacts were advised to visit the clinic if symptomatic at any time. Contacts with a normal CXR, but with persisting symptoms were evaluated for EPTB. The contacts who were diagnosed with TB disease were initiated on treatment as per national guidelines.

According to WHO guidelines [17], Pulmonary tuberculosis (PTB) refers to any bacteriologically confirmed or clinically diagnosed case of TB involving the lung parenchyma or the tracheobronchial tree. Extrapulmonary tuberculosis (EPTB) refers to any bacteriologically confirmed or clinically diagnosed case of TB involving organs other than the lungs, e.g. pleura, lymph nodes, abdomen, skin, meninges, genitourinary tract, joints and bones.

### Statistical analysis

SPSS 24 was used for data analysis. Categorical variables were described in terms of frequencies and percentages. The goal of the analysis was to determine whether characteristics of the HHC and environmental factors were associated with active TB in HHC as well as index cases. To determine factors associated with active TB, logistic regression analysis was performed, and predictor variables included clinical and epidemiologic characteristics of the HHC as well as environmental factors. Variables associated with TB in the univariate analysis (*p* <  0.05) were retained for the multivariate analysis. A 95% confidence interval and a 5% level of significance were used to interpret statistical significance. The *p*-values < 0.05 were considered significant.

## Results

### Household contacts

A total of 2397 HHC were living with 443 index cases with a median number of 5 contacts. Of these, 1321 (55.1%) were adults and 53.3% of overall contacts were recorded as females. Among 1076 (44.9%) children, 46.2% were aged ≤ 5 years. Overall literacy rate was very low among adults with 623 (47.2%) found to be illiterate with no formal education, and majority were females with no or little (just able to read and write) educational background.

### Screened household contacts

For 2397 HHC, 223 (9.3%) were screened based on symptoms. Seventy nine (35.4%) contacts had any cough, whereas 67 (30%) had chronic cough (more than a month) at the time of screening. Approximately 17 contacts with chronic cough did not give sputum due to dry cough and 6 refused to submit the sputum, which were mostly likely to be females. Of 223, 119 (53.4%) were females, 143 (64.1%) were children ≤ 14 (Table 1). Among children, 55.1% were aged ≤ 5 years. Forty five (20.2%) had changes in CXR but only 26 (11.7%) were observed with TB while the rest seemed to have other lungs related complexities. Amongst totally contacts screened, siblings including brothers, sisters and cousins reached the reached the top figure of 92 (41.3%).

**Table 1:** Characteristics of screened household contacts at the study site (*n* = 223)

Variables	No. (%)
Gender	
Male	104 (46.6)
Female	119 (53.4)
Age (years)	
≤ 14	143 (64.1)
> 15	80 (39.9)
CXR	
Normal	197 (88.3)
Abnormal	26 (11.7)
TST	
Positive (≥ 10 mm)	176 (57.4)
Negative (< 10 mm)	47 (42.6)
Weight (percentiles)	
Normal weight	27 (12.1)
Underweight^a^	196 (87.9)
Relationship to index case	
Mother	50 (22.4)
Father	33 (14.8)
Sibling^b^	92 (41.3)
Grandparent	34 (15.2)
Uncle/Aunt	14 (6.3)

*CXR* chest X-ray, *TST* Tuberculin Skin Test

^a^< 5 percentiles

^b^including brothers, sisters and cousins

### Secondary TB cases

Table 2 shows the characteristics of secondary cases. Contact tracing of the HHC of children with active TB allowed for the identification of an additional 35 TB cases among household members. Around 60% of these were females, 60% children ≤ 5 years and 77.1% had BCG present. A significant proportion of cases were noted as underweight (91.4%) with majority being from urban areas. More than half of the cases were reported for PTB-.

**Table 2:** Characteristics of secondary TB cases among the household contacts of children with active TB (*n* = 35)

Characteristics	No. (%)
Gender	
Male	14 (40)
Female	21 (60)
Age (years)	
≤ 5	21 (60)
6–14	9 (25.7)
15–44	4 (11.4)
≥ 45	1 (2.9)
Residence	
Rural	11 (31.4)
Urban	24 (68.6)
Weight (percentiles)	
Underweight^a^	32 (91.4)
Normal weight	3 (8.6)
BCG scar	
Present	27 (77.1)
Absent	8 (22.9)
Type of TB	
PTB+	7 (20)
PTB-	19 (54.3)
PTBNS	9 (25.7)

*PTB-* smear negative pulmonary TB, *PTB+* smear positive pulmonary TB, *PTBNS* pulmonary TB with unknown sputum

^a^< 5 percentiles

**Fig. 1:**
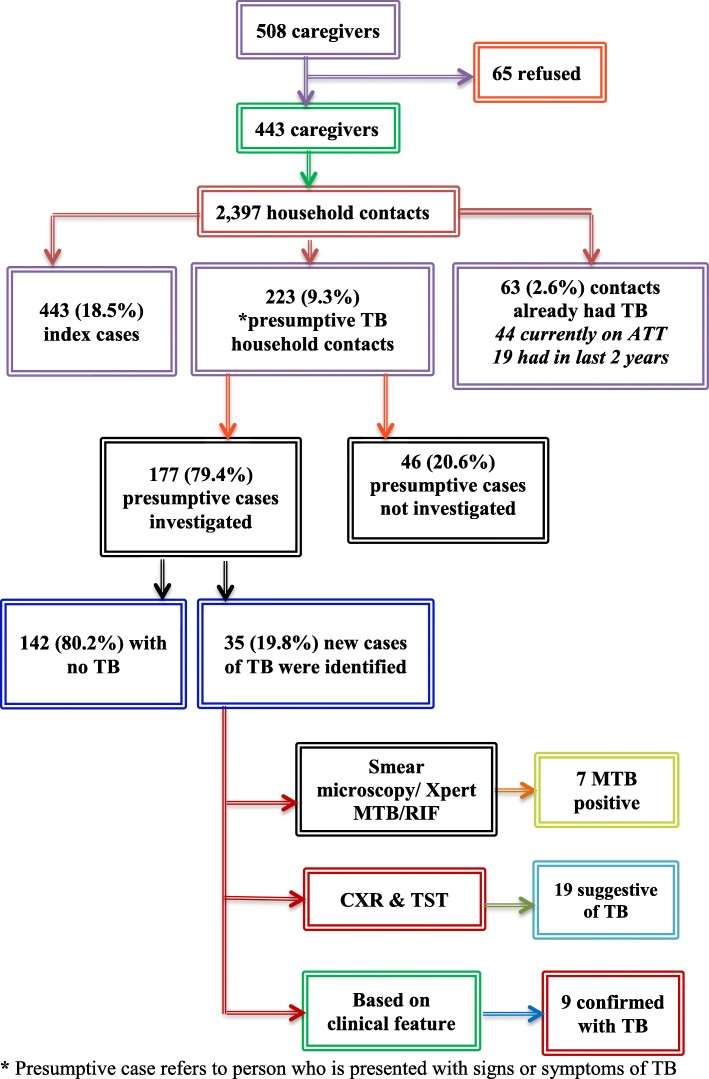
Screening of household contacts of index cases. * Presumptive case refers to person who is presented with signs or symptoms of TB

During screening, 7 (20%) HHC were diagnosed by sputum smear microscopy/Xpert MTB/RIF and CXR, 19 (54.3%) on the basis of CXR changes and TST positivity, while 9 (25.7%) were diagnosed on the basis of clinical features (Fig. 1). The identified cases were predominantly females (59.3%) and most of the cases belonged to age ≤ 14 years (74.1%). Of total adult cases, 2 had previous history of TB and were confirmed as default cases. About 29.6% of diagnosed cases did not present any clinical symptoms. Abnormalities on CXR included cavitation (3.7%), unilateral infiltration (25.9%) and bilateral infiltration (14.8%). BCG scar was present in 51.9% of cases.

### Characteristics of TB source cases for the study cohort

Around 63 (14.2%) of the index cases enrolled in the study had at least one other previously known TB case in the households (Table 3). Of these, more than half of the cases were identified for mothers and fathers (39.7 and 27%, respectively). A notable number of the cases (60.3%) were aged 15 to 44 years and 49.2% were found to have PTB+. Seven of the source cases had MDR-TB, 2 were recorded as default and 5 as failure cases.

**Table 3:** Characteristics of TB source cases (*n* = 63)

Variables	No. (%)
Relationship to index case	
Father	17 (27)
Mother	25 (39.7)
Sibling^a^	2 (3.2)
Grandparent	11 (17.5)
Uncle/Aunt	6 (9.5)
Relative	2 (3.2)
Age (years)	
≤ 5	00
6–14	2 (3.2)
15–44	38 (60.3)
≥ 45	23 (36.5)
Type of TB	
PTB+	31 (49.2)
PTB-	16 (25.4)
EPTB	5 (7.9)
No record^b^	11 (17.5)

*PTB-* smear negative pulmonary TB, *PTB+* smear positive pulmonary TB, and *EPTB* extra-pulmonary TB

^a^including brothers, sisters and cousins

^b^Record was not possible to find

### Risk factors of TB among the HHC of children with active TB

In univariate analysis (Table 4), children ≤ 2 years (OR = 2.647, 95% CI: 1.264–5.541), family income < 5000 PKR (OR = 5.935, 95% CI: 1.410–24.982), living in shelter (OR = 3.269, 95% CI: 1.557 to 6.862), HHC with TB (OR = 21.892, 95% CI: 8.432 to 56.849), contact of > 18 h with TB individual (OR = 6.091, 95% CI: 2.808 to 13.210), overcrowding in house (OR = 4.525, 95% CI: 51.331 to 15.390), HHC with smoking (OR = 10.929, 95% CI: 3.716 to 32.214), and drinking water without boiling (OR = 4.879, 95% CI: 1.436 to 16.572), had statistically significant positive association with TB.

**Table 4:** Univariate analysis of risk factors for type of TB among the screened HHC of children with active TB (n = 223)

Characteristics	TB		OR	95% CI	*P*-value
	Yes n (%)	No n (%)			
Gender					
Male	14 (13.5)	90 (86.5)	0.726	0.348–1.513	0.393
Female	21 (17.6)	98 (82.4)	1.378	0.661–2.871	
Age (years)					
≤ 2	21 (23.6)	68 (76.4)	2.647	1.264–5.541	0.010
3–5	9 (16.7)	45 (83.3)	1.100	0.480–2.519	0.822
6–10	4 (7.5)	49 (92.5)	0.366	0.123–1.090	0.071
11–14	1 (3.7)	26 (96.3)	0.183	0.024–1.397	0.102
Residence					
Rural	11 (12.9)	74 (87.1)	0.706	0.327–1.527	0.376
Urban	24 (17.4)	114 (82.6)	1.416	0.655–3.036	
Weight (percentiles)					
Underweight^a^	32 (16.3)	164 (83.7)	1.561	0.443–5.495	0.488
Normal weight	3 (11.1)	24 (88.9)	0.641	0.182–2.255	
BCG scar					
Present	27 (14.3)	162 (85.7)	0.542	0.222–1.320	0.177
Absent	8 (23.5)	26 (76.5)	1.846	0.757–4.500	
Monthly income of family (PKRs)					
< 5000	4 (50)	4 (50)	5.935	1.410–24.982	0.015
6000–10,000	21 (17.8)	97 (82.2)	1.407	0.675–2.933	0.362
11,000–20,000	8 (9.5)	76 (90.5)	0.437	0.188–1.012	0.053
> 20,000	2 (15.4)	11 (84.6)	0.975	0.207–4.603	0.975
Education of caregiver					
No formal education	19 (20.2)	75 (79.8)	1.789	0.865–3.699	0.116
Primary	3 (9.4)	29 (90.6)	0.514	0.148–1.790	0.296
Secondary	8 (19.5)	33 (80.5)	1.392	0.581–3.335	0.459
College	2 (7.7)	24 (92.3)	0.414	0.093–1.838	0.246
Graduation	3 (10)	27 (90)	0.559	0.160–1.954	0.362
Type of housing					
Slum house	3 (8.6)	32 (91.4)	0.457	0.132–1.584	0.217
House	9 (10.2)	79 (89.8)	0.478	0.212–1.075	0.074
Apartment	5 (13.9)	31 (86.1)	0.844	0.304–2.346	0.745
Shelter	18 (28.1)	46 (71.9)	3.269	1.557–6.862	0.002
Number of rooms in house					
1–2	26 (17.2)	125 (82.8)	1.456	0.644–3.294	0.367
3–4	9 (12.5)	63 (87.5)	0.687	0.304–1.554	
Overcrowding					
Members per household > 6	32 (19.5)	132 (80.5)	4.525	1.331–15.390	0.016
Members per household ≤6	3 (5.1)	56 (94.9)	0.221	0.065–0.752	
HHC with TB					
Yes	29 (46)	34 (54)	21.892	8.432–56.849	< 0.001
No	6 (3.8)	154 (96.2)	0.046	0.018–0.119	
HHC with smoking					
Yes	31 (28.4)	78 (71.6)	10.929	3.716–32.214	< 0.001
No	4 (3.5)	110 (96.5)	0.091	0.031–0.270	
Time spent with TB HHC (hours)					
8–11	5 (7.9)	58 (92.1)	0.374	0.138–1.011	0.053
12–17	7 (7.6)	85 (92.4)	0.303	0.126–0.728	0.008
> 18	23 (33.8)	45 (66.2)	6.091	2.808–13.210	< 0.001
Cooking done in the house					
Inside the kitchen	28 (15.2)	156 (84.8)	0.821	0.330–2.041	0.671
Inside the room	5 (21.7)	18 (78.3)	1.574	0.543–4.562	0.403
Outside the space attached to house	2 (12.5)	14 (87.5)	0.753	0.163–3.470	0.716
Kind of fuel used for cooking					
LPG	2 (15.4)	11 (84.6)	0.975	0.207–4.603	0.975
Natural gas	24 (16.2)	124 (83.8)	1.126	0.519–2.444	0.764
Wood	8 (23.5)	26 (76.5)	1.846	0.757–4.500	0.177
Charcoal	1 (3.6)	27 (96.4)	0.175	0.023–1.335	0.093
Use boiled milk					
Yes	6 (16.2)	31 (83.8)	1.048	0.401–2.736	0.924
No	6 (31.6)	13 (68.4)	2.785	0.980–7.912	0.055
Do not use milk	23 (13.8)	144 (86.2)	0.586	0.270–1.272	0.176
Use boiled water for drinking					
Yes	3 (4.8)	59 (95.2)	0.205	0.060–0.696	0.011
No	32 (19.9)	129 (80.1)	4.879	1.436–16.572	

^a^< 5 percentiles

In multivariable analysis (Table 5), variables which had statistically significant positive association in developing TB were HHC with TB (OR = 15.288, 95% CI: 5.378–43.457), HHC with smoking (OR = 7.094, 95% CI: 2.128–23.648), and contact of > 18 h with TB individual (OR = 4.681, 95% CI: 1.198–18.294).

**Table 5:** Multivariate analysis of risk factors for type of TB among the screened household contacts of children with active TB (n = 223)

Variables	β	SE	OR	95% CI	*P-* value
Age ≤ 2	0.659	0.493	1.933	0.735–5.085	0.181
Income < 5000 PKR	1.239	1.149	3.453	0.364–32.797	0.281
Living in shelter	0.825	0.506	2.281	0.847–6.145	0.103
Members per household > 6	0.557	0.747	1.745	0.404–7.544	0.456
Members per household ≤6	−0.557	0.747	0.573	0.133–2.477	0.456
Time spent 12–17 h	−0.331	0.791	0.718	0.152–3.386	0.676
Time spent > 18 h	1.543	0.695	4.681	1.198–18.294	0.026
HHC with TB	2.727	0.533	15.288	5.378–43.457	< 0.001
HHC with no TB	−2.727	0.533	0.065	0.023–0.186	< 0.001
HHC with smoking	1.979	0.610	7.094	2.128–23.648	0.001
HHC without smoking	−1.979	0.610	0.138	0.042–0.457	0.001
Using boiled water	−1.407	0.753	0.245	0.056–1.072	0.062
Using water without boiling	1.407	0.753	4.097	0.937–17.906	0.062

*β* beta, *SE* standard error, *CI* confidence interval, *OR* odd ratio

## Discussion

Population based screening has been by and large discouraged by the reason of high cost, low efficacy and poor feasibility. Nevertheless, screening the targeted risk group might be more practical and cost-effective [18]. In the present study, 35 (1.5%) secondary TB cases were diagnosed with TB including 30 children aged ≤ 14 years. Total 19 (54.3%) cases of PTB- and 7 (20%) cases of PTB+ were identified among adults and children. In present study, only 9.3% of contacts were screened and 15.7% of these were diagnosed with TB. The proportions are low and need to be improved in future. Studies have reported that up to 22% of HHC have TB in high prevalence countries [19]. The low rates of contacts screened is possibly a repercussion of the passive nature of the program, which mainly depend on distinctive clinical symptoms being experienced by the contacts [18]. However, the high rate has been reported in a study conducted in South Africa, where a thorough investigation of HHC irrespective of symptoms resulted in detection of 17.4% of new TB cases [20].

In the current study, a significant number of contacts did not go for screening even when they experienced sustained cough. Reported foremost reasons for this were unawareness in respect to the need for screening, illiteracy and fear of additional cost of diagnosis and treatment. Majority of caregivers did not know that contacts with prolonged cough be in need of screening. Likewise, only some of them realized that young children ought to be screened in case of suspected symptoms and consequently children especially < 5 years of age are usually overlooked. The overall prevalence of TB among HHC was 1.5%. These results are comparable to recently published study from India [5]. In another study conducted in Uganda, 6% detection rate was reported among the HHC of TB index cases [21] with greater number in children than adults which is line with our findings. Recently published a meta-analysis across Africa, Asia and Middle-East *has* recorded a prevalence of 0.1–6.2% among the contacts screened by way of contact investigation [22]. If TB screening would have done systematically for all children in this study, we estimate that twice as many TB cases could be recognized.

First degree relatives are up to 5 times more inclined to cause infection in HHC [23]. In the present study, for source case, 63 of HHC were identified with 44 for having TB at present and 19 had during the past 2 years. Around 96.8% of the confirmed household source cases were adults with highest being mothers (39.7%) followed by fathers (27%). Our results are in agreement with the study from India where majority of the source cases were identified for adults [24] and particularly for parents [25]. Our finding for majority of the source as a mother is in agreement with the review article where a number of studies have mentioned that children whose source case is a female family member are at greater risk [11]. Results of a study from Pakistan has also reported increased number of mothers as the contact of children with active TB [26]. The most proper clarification of this fact could be that in Pakistan, like in many parts of the world, younger children are in closer physical contact with their mothers than their fathers and spend more time at home in close contact with females than males. In addition, the degree of exposure with TB contacts was assessed by recording the closeness of patients to the individual with TB within the household (in terms of time spend with TB contact). Contact of > 18 h per day with TB individual was significantly connected with TB among HHC. In numerous cases, prolonged contact occurred when the child or household is dependent on the individual with TB.

The researchers of one study conducted in South Africa, observed that children who were diagnosed with TB on CXR seemed to be asymptomatic [27–29]. This shows that symptom based screening would miss some children with primary lung involvement on CXR. This study shows that the proportion of HHC of children with active TB screened under the current passive screening system of the NTP was very low. This is because the caregivers were asked to bring only the symptomatic contacts for screening. There could be possibility that not all the symptomatic contacts were attended at the treatment centres. In addition, there might be some asymptomatic contacts that could have TB infection. For that reason, the entire HHC of TB patient regardless of symptoms should be screened in order to have the early finding of additional cases of TB and to reduce TB transmission. This will increase the rate of detection for TB among the HHC of index cases and source case for child with TB.

Children, with a household source case and other caregivers especially grandparents or extended family members who take care of them were found at high risk of TB, which is in agreement with [30]. HHC of patient with TB have been reported at higher risk of infection than individuals in the general population. A number of modern studies conducted among children [31–33] further confirmed that contact with a TB patient appeared as the paramount risk factor for TB infection. A statistically significant association of TB contacts was observed with age and gender as greater number of contacts were seen for children aged ≤ 5 years and female patients in the newly identified TB cases among the HHC. Similar results are reported from the studies conducted in Pakistan and Gambia [34–36]. This shows the behavioural and cultural trend of study area where females (or girls) are confined to stay most of the times at home and less often get interaction with the people in community.

In the present study, host factors were not the exclusive factors associated with secondary cases of TB in HHC. Among the various environmental factors investigated, the cigarette smoke was found as significant risk factor of TB on multivariate analysis for the secondary TB cases. In accordance with WHO, smoking increases the risk of TB and > 20% of global TB incidence may be associated with smoking [37]. Exposure to tobacco smoke as significant predictors for TB infection among children of respective age has previously been reported by [38]. Furthermore, factors including children aged ≤ 5 years, overcrowding, living in shelter, and using water without boiling were observed as significant risk factors on univariate analysis.

Crowding reveals the increased likelihood of coming into contact with infectious persons expelling the bacilli in crowded environments supported by poor ventilation, recirculation as well as greater sharing of air [39]. These findings persisted in the current study where majority of patients (67.7%) were living in the houses with 1 to 2 rooms, 28.7% living in the shelters and 73.5% had > 6 family members reflecting the level of poverty and congested indoor environment. A study conducted in Pakistan, has presented the same level of living environment where 55% of children had to live in a single room house with families comprising 5 or more members [34]. Moreover, majority of these people were living in the areas with poor sterile conditions, poor cleanliness and more noteworthy communication among families and the neighbours which in turn increases the risk of getting and developing disease and infection at a more prominent rate. Howbeit, there is significance to HHC tracing, protocols must be confirming with the lifestyle of the target population. Frequent, intense contact with people outside the family is even more definite in crowded urban slums globally. In Pakistan, mostly children ride to school with 5 to 6 other children in an auto rickshaw designed for 3 on average. Public transports in these settings also carry far more people than there are seats. In the study area, children typically males aged 7 or more had practice to play outside in the streets most of the times which expose them to more contact in the community, hence making it difficult to identify the index case. Contact tracing procedures must therefore account for extent, closeness, and frequency of potential TB contacts further than household. Keeping all this in mind, the school-based TB-screening program at the study site would be beneficial in detecting early cases of TB. This strategy has been previously reported as cost-effective for detecting LTBI among children [40].

Giving the significance to screening contacts beyond the HHC, a study conducted in Pakistan in addition to 22.3% of TB prevalence among household has likewise watched extra 19.1% of predominance in close community [12]. As the strong evidence of close community investigation on TB case detection has been given by Aashifa et al.*,* once the cost adequacy of close community investigation is set up, and the achievability of execution in to routine exercises is considered, community-based screening may grow across the country by the national program. Interventions like household visits by the health workers can provide an opportunity to educate family members and the community about screening HHC. Community education may possibly help parents on how TB presents in children, essentiality of screening and encourages accessing the healthcare at proper time. Additionally, further strategies to improve documentation of possible contacts should be given due consideration to increase the yield of contact investigation. Active case finding strategy should be initiated to have maximum case detection in family and neighborhood.

## Conclusion

This study shows that the proportion of HHC of TB children screened under the current passive screening system of the NTP was very low. This is because the caregivers were asked to bring only the symptomatic contacts for screening. There could be possibility that not all the symptomatic contacts were attended at the treatment centres. In addition, there might be some asymptomatic contacts that could have TB infection. For that reason, the entire HHC of TB patient regardless of symptoms should be screened in order to have the early finding of additional cases of TB and to reduce TB transmission.

### Limitations

The present study has a few limitations. To start with, because of restricted human and money related assets screening was performed just in symptomatic contacts that may have brought about under-diagnosis. Second, the screening was constrained just to the HHC which implies the results cannot be summed up to the community or the neighbourhood. Despite the fact that, there is plausibility of finding the extra TB cases in the community as the quantity of cases were over and over found in some specific territories.

## Data Availability

The datasets used in this study are available from the corresponding author upon reasonable request.

## Abbreviations

CI: confidence interval
CXR: chest X-ray
*DOTS*: *Directly Observed Treatment Short* Course
EPTB: extra-pulmonary TB
HHC: household contacts
ICDK: Institute of Chest Diseases Kotri, Sindh
LUMHS-CHH: Liaquat University of Medical and Health Sciences Civil Hospital, Hyderabad
NTP: National TB Program
OR: odd ratio
PTB: pulmonary TB
PTB-: smear negative PTB
PTB+: smear positive PTB
PTBNS: pulmonary TB with unknown sputum
SBGH-LH: Shah Bhitai Government Hospital, Latifabad, Hyderabad
SBS-THM: Syed Baqadar Shah Taluka Hospital, Matiari
SE: standard error
SGH-QH: Sindh Government Hospital Qasimabad, Hyderabad
TB: tuberculosis
TST: Tuberculin Skin Test
Xpert MTB/RIF: Gene Xpert MTB/RIF assay
β: beta
